# Health literacy is much more than knowing about health; it also involves the emotions experienced during illness

**DOI:** 10.1371/journal.pdig.0000979

**Published:** 2025-08-07

**Authors:** Felipe Cezar Cabral, Maria Eulália Vinadé Chagas, Gabriela de Oliveira Laguna Silva, Guilherme Alcides Flores Soares Rollin, Mariana Giordano Cordoni, Lindayane Debom Motta, Leo Anthony Celi, Taís de Campos Moreira

**Affiliations:** 1 Digital Health, Hospital Moinhos de Vento, Porto Alegre, Brazil; 2 Medical Affairs, Kenvue, São Paulo, Brazil; 3 University of São Paulo, São Paulo, Brazil; 4 Institute for Medical Engineering and Science, Massachusetts Institute of Technology, Cambridge, Massachusetts, United States of America; Emory University, UNITED STATES OF AMERICA

Health literacy is the ability to understand health-related information, make informed choices, and act consciously to maintain or improve one’s health [1]. It is a key determinant of chronic disease management, as it empowers patients to engage in self-care and participate meaningfully in clinical decision-making. However, a significant challenge in this context is epistemic injustice, which occurs when patients’ knowledge, experiences, and emotions are undervalued or dismissed during clinical encounters [2]. This form of injustice can lead to poor self-management, even among individuals with adequate health literacy [1]. For type 2 diabetes mellitus (T2DM), which affects approximately 537 million people worldwide and is projected to increase by 46% by 2045 [3], interventions focused on improving health literacy, particularly those that also consider the emotional and subjective experiences of patients, are essential. Such interventions are not only cost-effective [4] but also associated with improved glycemic control, enhanced symptom management, and adoption of healthier lifestyle behaviors, including diet, physical activity, and medication adherence [5].

Given the importance of literacy in T2DM, digital health plays a key in supporting personalized self-care and education. It enables real-time glucose monitoring, behavioral data tracking, and provides personalized feedback, helping patients understand how daily actions influence glycemic control and overall well-being. These tools enhance self-care and foster behavior change [6–8]. To explore how health literacy intersects with emotional experiences in diabetes management, we conducted a mixed-methods study at a hospital in southern Brazil. The study included ten patients with T2DM and HbA1c ≥ 8%, recruited from the hospital’s outpatient diabetes clinic. It was conducted over 12 months in two phases. In the first phase, participants completed the validated Brazilian version of the Functional Health Literacy Test in Adults (TOFHLA) [9]. before and after six synchronous online educational sessions focused on diabetes self-care. The second phase was facilitated by a nurse and a psychologist, the sessions included a focus group where participants discussed their diabetes management experiences and emotional challenges.

The initial assessment showed all participants had adequate health literacy, which remained unchanged after the intervention. Consequently, focus group discussions shifted toward the emotional and psychological aspects of living with diabetes. Patients shared that the classes helped reinforce forgotten knowledge and self-care behaviors, and emphasized the value of peer support and multidisciplinary guidance in enhancing motivation, emotional well-being, and effective disease management.

The sharing of daily challenges, such as medication adherence, dietary management, physical activity, and coping with complications, highlighted that periodic consultations alone are insufficient to sustain effective diabetes management. A broader, integrated approach is needed, where a multidisciplinary team complements medical care by addressing emotional, psychological, and behavioral needs. In our experience, the multidisciplinary team included a psychologist, nurse, and nutritionist, who provided support not only during the focus group but also throughout the educational intervention. This support fostered emotional well-being, motivation, and patient engagement in diabetes self-care. These reflections reinforce the need for diabetes care that addresses both cognitive and emotional aspects of management. Even individuals with adequate health literacy may face challenges in consistently practicing effective self-care when emotional distress, feelings of being unheard, or relational barriers with healthcare providers are present. Recognizing and validating these emotional dimensions is essential to support behavior change and promote more effective and sustainable diabetes self-management.

The focus group also allowed us to explore how patients experience epistemic injustice, particularly when their emotions or knowledge are dismissed in clinical encounters. These discussions revealed how digital health can help surface such issues by creating new spaces for patient expression and recognition. Thus, beyond promoting education and engagement, digital tools also offer pathways to identify and address forms of epistemic exclusion that often go unnoticed in traditional healthcare settings [6–8].

Based on these reflections, we propose four key recommendations to improve diabetes care. It is important to note that digital health is not an isolated intervention but rather a transversal enabler that supports and amplifies all these recommended actions. Whether facilitating patient education, enhancing communication, or supporting behavior change, digital tools play a critical role in promoting health literacy, fostering epistemic humility, and improving patient navigation.

**1. Assess the level of health literacy as part of the patient history.** This allows clinicians to adjust communication and care to each patient’s needs [10].
**2. Foster epistemic humility as a foundation for clinical practice.** Respecting patients’ beliefs, perspectives, and judgments allows clinicians to achieve a more accurate and enriching understanding of their reality, promoting truly patient-centered care [11].
**3. Empower patients through health navigation and psychosocial support.** This provides assistance and guidance that help manage the complex demands of diabetes, adopting a person-centered and holistic approach [12,13].
**4. Expand research and training on health literacy and digital health literacy.** This includes building capacity among healthcare teams and evaluating how health systems can better support patients with varying levels of literacy [14].

Digital health solutions play a crucial role in enabling these strategies. When thoughtfully integrated, digital tools can amplify patient voices, offer tailored education, create new channels for communication, and help surface and mitigate epistemic exclusion in clinical care. However, this potential will only be realized if digital solutions are designed with inclusivity in mind and address barriers related to digital literacy especially among older adults, rural populations, and individuals with limited connectivity or low levels of digital or health literacy [6–8,15].

Furthermore, the successful management of diabetes, whether within telemedicine contexts or traditional care models, requires both patients and healthcare professionals to embrace epistemic humility. It is crucial to recognize the inherent asymmetry in medical encounters, where clinicians often hold greater epistemic authority, influencing patient decisions and care trajectories. This relationship is neither neutral nor egalitarian. Social determinants of health deeply shape how individuals access information, interpret medical advice, and engage in self-care, as well as how their knowledge and experiences are valued within clinical settings. Ultimately, approaches that integrate health and digital literacy, while addressing emotional, cognitive, and social dimensions, can improve diabetes care and quality of life for people with T2DM.
